# Ataxia and Hypothyroidism

**Published:** 1965-01

**Authors:** Bryan Ashworth

**Affiliations:** Senior Medical Registrar, Royal Infirmary, Bristol


### ATAXIA AND HYPOTHYROIDISM

BY

BRYAN ASHWORTH, M.B., M.R.C.P., M.R.C.P.E.

*Senior Medical Registrar, Royal Infirmary, Bristol*

The purpose of this paper is to draw attention to the occurrence of ataxia as a presenting symptom of hypothyroidism. Five of the cases here reported have been seen in one Neurological Clinic during the past two years. The patients in this series were middle aged or elderly and the ataxia had sometimes been attributed to vascular disease (which was often present) or degenerative disease of the cerebellum. In younger patients the diagnosis of disseminated sclerosis might be considered or the symptoms be regarded as psychogenic in origin.

The term myxoedema is applied to a severe degree of hypothyroidism which can be diagnosed at sight by the experienced clinical observer. Because this is widely recognized there is a tendency for the diagnosis of hypothyroidism to be overlooked in patients who show neither the characteristic symptoms nor the classical signs of myxoedema. Yet the treatment of mild forms of hypothyroidism is both simple and rewarding.

### HISTORY

The occurrence of staggering and awkwardness of movement in myxoedema was well known to the Committee of the Clinical Society of London which investigated a series of patients in 1888 before any specific treatment was available. Incoordination was found in 39 of 59 patients.

The neurological aspects were discussed by Söderbergh (1912) who thought that the slowness of movement and speech in myxoedema were not due to mental torpor but indicated a disturbance of cerebellar function. He described clumsiness, vertigo, tremor in attempting to pick up small objects, writing like that found in cerebellar disorders, adiadochokinesia and cerebellar catalepsy. He found that all these abnormalities cleared with treatment of myxoedema.

Söderbergh observed that percussion of a muscle such as biceps in a patient with myxoedema caused a transverse swelling to appear which persisted for up to six seconds. He also reported briefly a post mortem examination of a patient who had died in coma. There was atrophy of the brain and its coverings. The cerebellum was gelatinous in appearance.

Jellinek and Kelly (1960) reported six cases of a cerebellar syndrome in myxoedema. Five had an appearance which suggested myxoedema. Three patients showed a complete remission after adequate treatment with thyroid, two others improved with treatment and one was thought unsuitable for treatment because of angina pectoris. The serum cholesterol was normal in one patient with severe myxoedema but raised in four others (range 360–885 mg per cent). The cerebrospinal fluid protein was examined in three patients and found to be 150 mg per cent, 56 mg per cent, and 80 mg per cent respectively.

### CASE REPORTS

*Case 1*

A man of 70 years (BRI 002031) was seen in September 1963. He complained of difficulty in walking with swaying from side to side, present for a year but much worse during the past month. His gait was ataxic, the knee and ankle jerks were diminished, the plantar responses flexor, and ataxia was more marked on the left side. He was slightly deaf. BP 130/90. Two months later he was admitted for investigation and by that time his gait had deterioriated and it was difficult for him to walk. His appearance suggested hypothyroidism, his skin was dry, tendon jerks were delayed, dysarthria and dysphonia were present, there was bilateral dysdiadochokinesia and the thyroid gland was small. Investigation showed a protein-bound iodine level of 1.9 μg per cent (normal 4.0 to 8.0 μg per cent), serum cholesterol 210 mg per cent, cerebrospinal fluid contained no cells but protein 62 mg per cent, tanned sheep red cell agglutination test positive at 1/25,000. The ECG showed low voltage and flattening of T waves. Treatment was started with l-thyroxin 0.05 mg daily and the dose gradually increased. There was a striking improvement in the gait during the subsequent six months. After a year there was minimal ataxia.

*Case 2*

A woman of 54 years (BRI 006069) was seen in January 1963. She complained that her legs were weak and heavy and said that for nine months she had tended to drag the left foot. She also reported transient episodes of double vision on looking straight ahead, but only when writing 'everything goes far away'. For three months she had tended to drop things held in the hand. On examination she was obese and slow in thought and movement. The skin was dry. BP: 130/80. The gait was ataxic and there was clumsiness of all limb movements. Dysdiadochokinesia was marked on both sides and there was bilateral abnormality of the finger-nose and heel-knee tests. Speech was dysphonic; there was no nystagmus. Tendon jerks were delayed and plantar responses flexor.

*Investigations*

$I^{131}$ uptake: 20 per cent at four hours and 40 per cent at 72 hours. Protein-bound iodine at 48 hours 0.06 per cent of dose/litre. Serum cholesterol 360 mg per cent. CSF normal with protein 41 mg per cent. Urinary 17 hydroxysteroids 16.3 mg/24 hours. ECG and EEG normal. Air encephalogram normal. Tanned sheep red cell agglutination negative.

Treatment was started with thyroxin and there was a steady improvement. In January 1964 slight ataxia was still present and the tendon jerks delayed; the dosage of l-thyroxin was then 0.3 mg daily. It was increased to 0.4 mg and a few months later she reported further improvement.

In this case the association of double vision with ataxia at first raised the possibility of disseminated sclerosis. The diplopia however was transient and recurrent and present only on looking straight ahead when writing. This suggested that it was due to disturbance of convergence of the eye; it cleared with treatment.

*Case 3*

A woman of 75 years (BRI 045807), a teacher of the piano, was seen in January 1964. She complained of unsteadiness in walking and episodes of dizziness and vomiting which had been present for nine months. She had fallen several times. She was unable to sing or play the piano. She also reported that her hair was falling out, she tired easily, and she was more sensitive to cold. On examination she was obese, the skin was dry and the face puffy. She was mentally slow. BP 150/95. The gait was grossly ataxic, there was bilateral dysdiadochokinesia, and bilateral impairment in the finger-nose test. The tendon jerks were all delayed.

*Investigations*

Protein-bound iodine 6.0 μg per cent. Serum cholesterol 260 mg per cent. ECG normal.

Treatment with thyroxin was started and there was a steady improvement.

*Case 4*

A woman of 64 years was seen in April 1964 (BRI 053734) and complained of unsteadiness in walking. The symptoms had been present for three months and there had been steady deterioration. On examination the skin was dry and the face puffy, BP 140/80, there was ataxia and bilateral dysdiadochokinesia.

*Investigations*

Protein-bound iodine 5.2 μg per cent. Serum cholesterol 375 mg per cent. ECG normal.

Treatment was started with thyroxin. Three months later she reported an improvement in walking.

*Case 5*

A woman of 56 years was seen in July 1964 (BRI 059908). She complained of unsteadiness in walking and pains in the limbs. She had been well until 2 years previously apart from a chronic discharging ear. The ear continued to discharge and in 1963 a mastoidectomy was performed. Subsequently the ear remained dry. About 9 months before she attended she began to complain of dizziness and weakness in the limbs. Occasionally her left leg gave way. It had been noticed also that her memory was poor and her hair tended to fall out.

When seen she showed gross ataxia of gait and was unable to walk without support. Her appearance suggested hypothyroidism. Voice soft, hoarse, and slightly dysarthric. BP 130/90. No nystagmus. Finger-nose test well performed but slight ataxia in the heel-knee test. Bilateral dysdiadochokinesia. Sensation normal. Tendon jerks all delayed. The serum cholesterol was 270 mg per cent and the protein-bound iodine 1.8 $\mu g$ per cent. ECG showed sinus bradycardia, low voltage complexes and T wave inversion. The cerebrospinal fluid pressure was 135 mm and the fluid contained less than 1 cell/cu mm and protein 45 mg per cent, WR negative and Lange 0022000000. EEG showed a generalized slowing of cortical rhythm. Tanned red cell agglutination negative.

Treatment was started with thyroxin. After 2 weeks there was a striking improvement in the gait. After 3 weeks she could walk without support but was still ataxic when turning.

*Case 6*

A woman of 60 years was seen in 1957 (BRI 058782). She complained of unsteadiness in walking with a tendency to sway to the left for 3 years. For six months she had noticed tiredness and increased sensitivity to cold. For six weeks she had complained of transient blurring of vision. It was noted that she tended to sway backwards when standing and the gait was ataxic. The serum cholesterol was 330 mg per cent, blood count normal, and the cerebrospinal fluid contained no cells but protein 56 mg per cent. Treatment with thyroid was started and within 3 weeks a marked improvement in the gait was noticed.

*Case 7*

A woman of 64 years was seen in 1960 (BRI 107035). She complained of difficulty in walking. This had first become apparent when she fell in the street 18 months previously. Her appearance suggested myxoedema, she was mentally slow and the gait was ataxic. The skin and hair were dry and the voice thick. The serum cholesterol was 290 mg per cent and the $I^{131}$ uptake 7 per cent at 4 hours. Blood count and ECG were normal. Treatment was started with thyroid and the gait improved. Later she became depressed and was treated in the Psychiatric Department. The gait continued to improve and she was followed up for 4 years.

### DIAGNOSIS

Ataxia was a presenting symptom in all these patients. In general the ataxia of gait was much more striking than might have been anticipated from examination on a couch. Difficulty in performing rapidly repeated movements was constantly present, dysarthria and dysphonia often occurred together, impairment of the finger-nose and heel-knee tests was never more than slight and nystagmus was not found. There was no disturbance of the motor system.

In the diagnosis of hypothyroidism a delayed tendon jerk was of the greatest value. It was often detected when eliciting the supinator jerk or the ankle jerk. In the more severe cases the tendon jerk was so much delayed that it might be regarded as absent. The delayed tendon jerk could be observed as a two phase relaxation of the muscle and a similar effect was produced if the muscle was percussed directly. This sign is sometimes called Woltman's sign in the United States and was described in detail 40 years ago (Chaney 1924).

Of the biochemical tests the protein-bound iodine estimation is the most specific. The serum cholesterol may be raised but a normal level is not a point against the diagnosis of hypothyroidism. The same may be said of the electrocardiograph which may be normal in an undoubted case of hypothyroidism. The level of protein in the spinal fluid may be raised as in Case 1 and in three cases reported by Jellinek and Kelly (1960).

The ultimate criterion in the diagnosis of hypothyroidism is the response to administration of thyroid (Wayne 1960). In assessing the response a clinical photograph is of great help but the state of the tendon jerks is also useful, particularly in mild degrees of hypothyroidism.

### PATHOLOGY

Little is known of the basic mechanisms which are responsible for this ataxic syndrome. No detailed histological studies have been reported. The age incidence suggests that vascular factors may be important. Arteriosclerosis cannot be more than a contributory factor since improvement or remission usually occurs with treatmen[illegible] A reduced cardiac output is known to occur in myxoedema (Davies, Mackinnon an[illegible] Platts 1952) and this may contribute (Bradshaw and McQuaid 1963).

### TREATMENT

The most useful preparation is l-thyroxin (Macgregor 1961) and is preferred t[illegible] thyroid extract because of its constant composition. The initial dose has usually bee[illegible] 0.05 mg daily and some improvement is usually apparent after a week. The dose i[illegible] increased only gradually with an interval of several weeks between increments i[illegible] dosage. The state of the tendon jerks is again helpful in assessing dosage.

### SUMMARY

Seven cases of hypothyroidism are reported in which ataxia was a presenting featur[illegible] While some showed the classical features of myxoedema the diagnosis of hypo-thyroidism was often not obvious. The presence of delayed tendon jerks was the mos[illegible] useful clinical sign of hypothyroidism and was present in all the cases. In all th[illegible] patients the ataxia improved with treatment with l-thyroxin. The importance o[illegible] recognizing hypothyroidism as a cause of ataxia is stressed.

I am grateful to Dr. A. M. G. Campbell for allowing me to describe these patients.

### REFERENCES

Bradshaw, P., McQuaid, P. (1963) "The Syndrome of Vertebro-basilar Insufficiency"[illegible] *Quart. J. Med.*, **32,** 279–297.

Chaney, W. C. (1924). "Tendon Reflexes in Myxoedema; a Valuable Aid in Diagnosis"[illegible] *J.A.M.A.*, **82,** 2013.

Davies, C. E., Mackinnon, J., Platts, M. M. (1952). "Cardiac Output in Myxoedema". *B.M.J.*[illegible] **ii,** 595–597.

Jellinek, E. H., Kelly, R. E. (1960). "Cerebellar Syndrome in Myxoedema". *Lancet*, **ii,** 225[illegible]

Macgregor, A. G. (1961). "Why does Anybody Use Thyroid B.P." *Lancet*, **i,** 329.

Söderbergh, G. (1912). "Symptômes Cérébelleux dans le Myxoedème." *Nord. Med. Arkiv*[illegible] **11,** 3.

Wayne, E. J. (1960). "Clinical and Metabolic Studies in Thyroid Disease". *B.M.J.*, **i,** 78.

Report of a Committee of the Clinical Society of London to investigate the subject of Myxoe-dema. *Clinical Society Transactions* (1888), vol. 21, suppl. 211.

